# Photoremoval of Bisphenol A Using Hierarchical Zeolites and Diatom Biosilica

**DOI:** 10.3390/ijms24032878

**Published:** 2023-02-02

**Authors:** Jagoda Chudzińska, Bartosz Woźniak, Myroslav Sprynskyy, Izabela Nowak, Agnieszka Feliczak-Guzik

**Affiliations:** 1 Faculty of Chemistry, Adam Mickiewicz University in Poznań, Uniwersytetu Poznańskiego 8, 61-614 Poznań, Poland; 2 Faculty of Chemistry, Nicolaus Copernicus University in Toruń, 7 Gagarina Str., 87-100 Toruń, Poland

**Keywords:** photocatalytic removal, photocatalysts, bisphenol A, hierarchical zeolites, diatom biosilica

## Abstract

Bisphenol A (4,4-isopropylidenediphenol, BPA) is an organic compound widely used, e.g., in the production of epoxy resins, plastics, and thermal receipt papers. Unfortunately, bisphenol A has negative effects on human health, which has prompted the search for an effective method of its removal. One of the most promising methods of its elimination is photocatalytic removal. The aim of this study was to design an effective method for the photocatalytic removal of bisphenol A using, for the first time, hierarchical zeolites and ruthenium ion-modified diatom biosilica, and silver as photocatalysts and optimization of the reaction conditions: temperature, pH, and composition of the reaction mixture as well as the electromagnetic wavelength. Additionally, for the first time, the electromagnetic wavelength that would be most suitable for the study was selected. All materials used were initially characterized by XRD and low-temperature nitrogen adsorption/desorption isotherms. Ruthenium ion-modified biosilica proved to be the most effective catalyst for bisphenol A removal, which occurred at a rate higher than 99%.

## 1. Introduction

Bisphenol A (4,4-isopropylidenediphenol, BPA) is a compound used in great amounts in the industry as a stabilizing monomer in the production of polycarbonate plastics, epoxy resins, and flame retardants. Among other things, it can be found in thermal receipt papers, beverage containers, powder coatings, medical equipment parts, and even electrical part covers [1,2,3,4].

It is stable at room temperature and has a mild odor reminiscent of chlorophenol. Thanks to the hydroxyl and methyl groups it contains bisphenol A and can actively participate in reactions, both substitution and hydrogenation, in different environments [5,6].

Unfortunately, this compound can exhibit negative effects on human health through its disruptive effects primarily on the endocrine system. According to literature reports, both bisphenol A and its analogs (bisphenol B, bisphenol S, bisphenol F) exhibit estrogenic effects, causing endocrine disruption, development of diabetes, asthma, heart disease, and even cancer [7]. In addition, this compound, found in the composition of food packaging, specifically canned food and beverage cans, may also be released into food products. This may be very dangerous to human health, given that the gastrointestinal tract is the largest source of absorption of substances [8]. As a result of this negative effect on human health, bisphenol A is counted among the compounds of considerable controversy, prompting numerous studies on its effective degradation. A prominent method that enables the effective degradation of bisphenol A is photocatalytic degradation, which involves the efficient oxidation of organic pollutants under the conditions of an available light source and a semiconductor that acts as a photocatalyst. Photoexcitation of the photocatalyst produces a jump of electrons from the valence band to the conduction band upon the absorption of radiation of a given energy. As a result of photoexcitation, an electron with reducing properties is generated in the conduction band, and a hole with oxidizing properties in the valence band [9]. In addition, photocatalysis stands out from other methods because of its environmental friendliness. It is also very inexpensive, does not require any additional chemicals to carry it out, and is quite easy to perform under aqueous conditions [10,11].

Using light energy and in the presence of an oxidant (oxygen), this method enables the complete decomposition of organic compounds into simple and atoxic products such as carbon dioxide CO_2_, water H_2_O, and other non-complex inorganic compounds. This technique is ideal for the degradation of even the most refractory organic pollutants. However, in order to optimize the conditions of the reactions carried out, external light sources besides sunlight are also used, generating visible light or/and ultraviolet radiation. The presence of photocatalysts is necessary, as they collect the supplied light and significantly contribute to the breakdown of organic compounds into simple molecules [10,11,12].

The photocatalytic degradation reaction may be used to purify both aqueous and gas environments. The reaction is based on the acceleration of the photoreaction taking place in the environment with the use of a suitable semiconductor, which is the photocatalyst of the reaction. The most commonly used photocatalysts are titanium(IV) oxide TiO_2_, zinc(II) oxide ZnO, cadmium sulfide CdS, tin sulfide ZnS, and iron(III) oxide Fe_2_O [13,14].

The photocatalytic degradation reaction begins with the adsorption of photons by a photocatalyst emitted from a light source (mainly ultraviolet radiation). Photons involved in the reaction should have a wavelength equal to or greater than the bandgap energy of a selected semiconductor. The effect of irradiation is the high-energy excitation of electron pairs of the photocatalyst and their subsequent displacement, resulting in:

- electrons in the conduction band initially coming from the valence band (eCB- electron in the conduction band). They act as very good reductants, e.g., reducing molecular oxygen to an anion radical O_2_^.^-. On continuing the reaction, it is possible to obtain hydroxyl radicals having one of the highest oxidation potentials;
- hole in the valence band (hVB+), formed when the electrons pair of eCB- disconnects and gain a positive charge. In the reaction, holes act as an oxidant, interacting with organic compounds undergoing degradation, leading to the final yield of mineralized products including CO_2_ and H_2_O. The oxidation process can also occur through the reactions with water, resulting in the formation of an electrophilic hydroxyl radical, which is involved in the oxidation of organic pollutants [13,15].

The resulting fragments are sequentially separated from one another, and then oxidation and reduction processes take place using hVB+ and eCB-. It is worth noting that both processes take place on the surface of the photocatalyst. Subsequently, there is the oxidation of organic compounds and the mineralization processes. One of the requirements for the reaction is the presence of molecular oxygen, which affects significant inhibition of the recombination process of the photocatalyst, so that the reaction may proceed. The recombination process takes place on the surface and in the volume of the semiconductor. Among the most important factors supporting a given process are impurities and any other particles affecting the changes in the volume or surface of the semiconductor [13,15].

The course of photocatalytic degradation is also significantly influenced by the pH value at which the process is carried out. This is due to the later charge of the photocatalyst, which can be both protonated and deprotonated. According to the literature, an acidic environment is preferred for a given reaction, characterized by a pH slightly below 7. In a given reaction environment, the surface of the photocatalyst becomes positively charged, supporting the adsorption of negatively charged organic compounds and contributing to an increase in the photocatalytic activity of the catalyst. In contrast, the presence of a negative charge on the surface would entail a decrease in Coulomb repulsion interactions and in photocatalytic activity. The most suitable temperature for the process is between 20 °C and 80 °C. A decrease in temperature supports the process of adsorption of impurities on the surface of the catalyst, while an increase in temperature disturbs adsorption. At temperatures close to 0 °C, the activation energy increases. As far as the concentration of the photocatalyst is concerned, it is evident that as it increases, the active surface area increases. This increase favors the generation of hydroxyl radicals and superoxide anion radicals, which are actively involved in the mineralization of organic compounds. Therefore, the same products may be obtained in less time and with greater yields. Unfortunately, when the photocatalyst concentration in the reaction mixture is too high, the reaction will noticeably slow down because of the possible formation of agglomerates blocking the light penetration to the affluent. Additionally, a too-low concentration of the photocatalyst will not benefit the photocatalysis process, as the amount used will most likely be insufficient to complete the process [13,16]. In this study, for the first time, hierarchical zeolites and diatom biosilica have been used as photocatalysts. Hierarchical zeolites are a special group of compounds characterized by a unique arrangement of pores and the presence of secondary porosity at the meso and macroscale. The peculiar arrangement of pores improves zeolite properties, i.e., imposes much smaller spherical constraints and improves the effectiveness of its photocatalytic performance, enabling processes with larger particles such as those of organic compounds [17].

Diatom biosilica is used for the adsorption of various pollutants and organic dyes from their aqueous solutions [18,19]. It has unique properties such as biocompatibility, mechanical and thermal stability, ordered structure, and valuable optical properties. It is also used in optical-electronic devices, as reaction catalysts, at water treatment plants for filtration processes [20,21].

The aim of this study was to develop an effective photocatalytic method for bisphenol A removal using hierarchical zeolites and diatom biosilica modified with silver ions and ruthenium used as photocatalysts. In the process of optimization of the photocatalytic bisphenol A removal, the impact of the composition of the reaction mixture, the pH of the reaction mixture, the range of electromagnetic wavelengths, and the temperature were examined. According to earlier studies, diatom biosilica has an openwork 3D structure with an ordered periodical pore network and shows unique optical properties (photonic), high thermal stability, and mechanical strength. Silica frustules isolated from diatom cell culture show high photoluminescence upon excitation with UV radiation in the blue (450–495 nm) and green (498–525 nm) range of visible light [20].

For example, TiO_2_-modified diatomaceous biosilica has been used as an effective photocatalyst for indoor air purification, for the photocatalytic degradation of rhodamine B as a catalyst for the photodegradation of the dye methyl blue, and as a photocatalyst in the reduction of acetaldehyde [14].

The crystal structure of zeolites (microporous materials), the negative charge of their crystal lattice and the ease of ion exchange outside the lattice, the uniform size of micropores, and high thermal and hydrothermal stability have led to the widespread use of these materials in many processes, including ion exchange, catalysis, oil processing, construction, purification processes, gas separation, and many others. The large-scale synthesis of zeolites as solid acid catalysts and the discovery of new materials have revolutionized the petrochemical and chemical industries. The microporosity of zeolites is a significant limitation to their applications, both in adsorption and catalysis. To overcome this limitation new hierarchical zeolites have been developed, exhibiting secondary porosity, that is, the presence of at least one additional pore system, primarily in the mesopore range. The presence of mesopores facilitates the access of large reactant molecules to the active centers of zeolites while maintaining their acidity and crystal structure [17].

The admixture of this material with active chemical elements such as noble metals, including Au, Ag, Ru, and Pd, can significantly affect the range and intensity of biosilica photoluminescence. Noble metal nanoparticles are a novel candidate for high absorption of visible light owing to their strong optical absorption in the entire solar region. The interesting aspect of metallic nanostructures correlates with their unique optical response that can be modulated upon variations of their specific size and morphology [22,23,24,25].

## 2. Results

### 2.1. Characteristics of Materials

**Figure 1:**
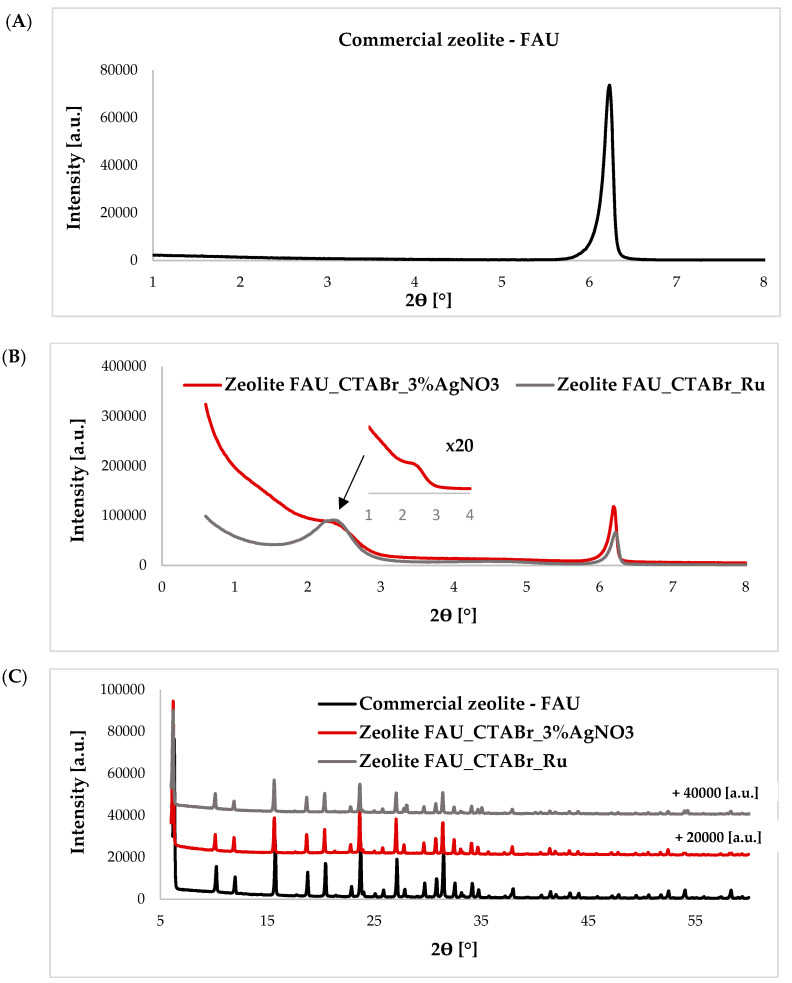
Diffractograms of (**A**) FAU-type commercial zeolite in the low-angle range, (**B**) hierarchical materials derived from FAU-type commercial zeolite in the low-angle range, (**C**) hierarchical materials based on FAU-type commercial zeolite in the wide-angle range.

Figure 1A shows the diffractogram of a commercial FAU-type zeolite in the low-angle range, while Figure 1B presents diffractograms of hierarchical zeolites modified with silver ions and ruthenium ions. From the diffractograms shown in Figure 1B, it can be concluded that additional porosity in the mesoporous range has been introduced, as evidenced by a reflection at an angle of 2θ ~ 2.5°. This reflection is absent in the diffractogram of the FAU-type commercial zeolite (Figure 1A). In addition, the structure of the commercial zeolite was preserved, as confirmed by the wide-angle diffractograms (Figure 1C).

**Figure 2:**
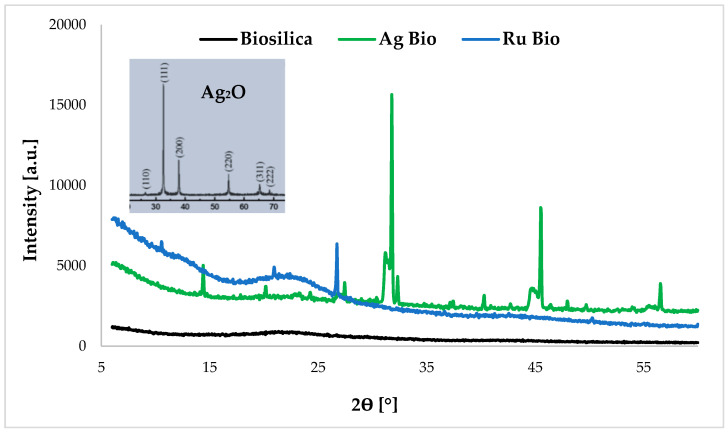
Diffractogram of porous materials based on diatom biosilica doped with silver or ruthenium ions in the wide-angle range.

In the diffractograms of materials obtained based on diatom biosilica (Figure 2), characteristic reflections assigned to Ag_2_O were observed for the material modified with silver ions [26]. In turn, the wide-angle diffractograms of the pure unmodified material and the material modified with ruthenium ions showed a characteristic reflection at 2θ = 22.3°, indicating the amorphous nature of the biosilica. According to Sprynskyy et al. [20], this diffractogram is characteristic of biogenic amorphous silica. The diffractogram for the material modified with ruthenium ions (Ru Bio) shows signals characteristic of ruthenium(IV) oxide nanoparticles. Analogous signals were obtained in our previous studies [17].

**Figure 3:**
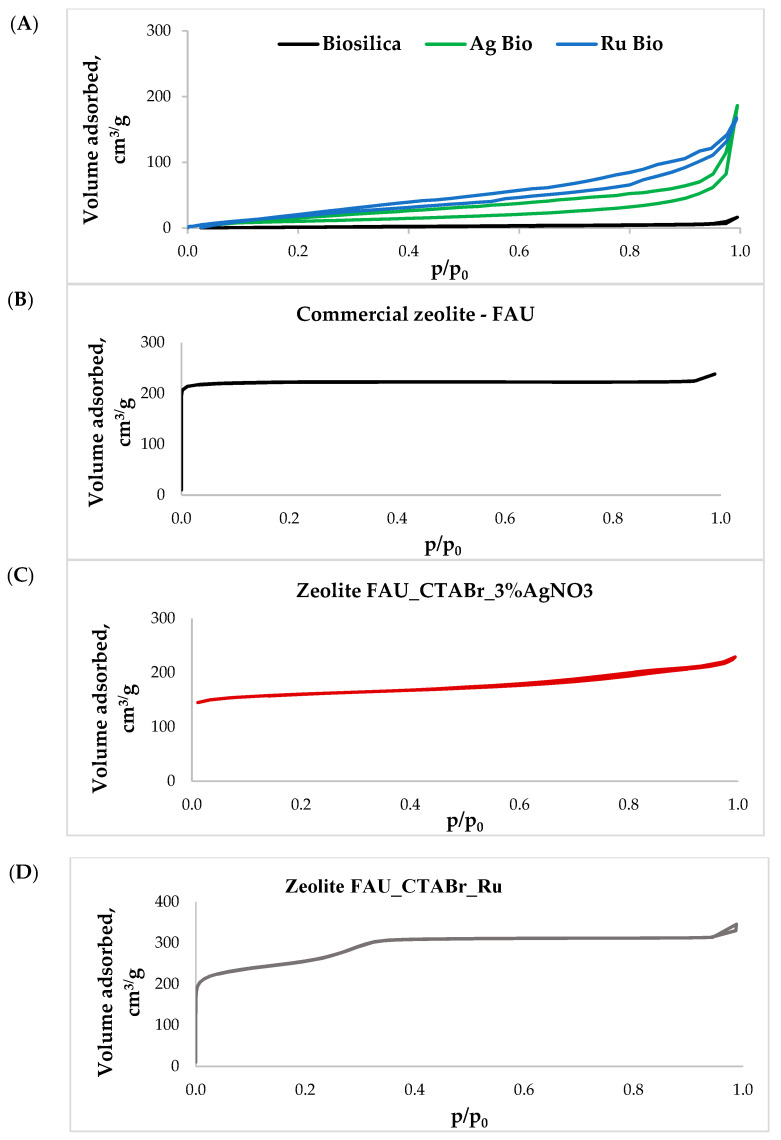
Nitrogen adsorption/desorption isotherms for (**A**) porous materials based on diatom biosilica doped with silver or ruthenium ions, (**B**) FAU-type commercial zeolite, (**C**) for hierarchical zeolite based on FAU-type commercial zeolite modified with silver ions, (**D**) hierarchical zeolite based on FAU-type commercial zeolite modified with ruthenium ions.

Figure 3 shows nitrogen adsorption/desorption isotherms for the materials studied. Figure 3A shows nitrogen adsorption/desorption isotherms for the materials obtained based on unmodified diatom biosilica, modified with silver ions or ruthenium. According to the IUPAC classification, the obtained adsorption/desorption isotherms belong to combinations of type I and II ones [27]. Type I is characteristic of microporous materials with relatively small external surfaces, while type II corresponds to nonporous or macroporous materials. In addition, the isotherms recorded for these materials show an H4-type hysteresis loop, indicating the presence of simple gap pores [28].

The nitrogen adsorption/desorption isotherms recorded for a commercial material of FAU type (Figure 3B) were found to be of type I according to IUPAC (International Union of Pure and Applied Chemistry), which is characteristic of microporous materials [28]. The isotherms obtained for the modified hierarchical materials were combinations of type I and type IV [27]. Type IV isotherm, according to the IUPAC classification, is characteristic of mesoporous materials. The results obtained (Figure 3C,D) confirm that the materials had a hierarchical structure with secondary porosity in the range of mesopores, which agrees with the results previously obtained by Feliczak-Guzik and co-workers [17,29,30].

Table 1 shows the values of textural parameters such as the specific surface area, pore volume, and average pore diameter obtained based on the isotherm data. The specific surface area of the materials was determined using the BET (Brunauer–Emmett–Teller) method. Pore size distributions were calculated using the KJS (Kruk–Jaroniec–Sayari) method [31] based on the BJH (Barrett–Joyner–Halenda) algorithm.

The materials: Biosilica, Ag Bio, and Ru Bio have relatively low surface areas (BET), from ~30 m^2^/g for pure diatom biosilica to ~104 m^2^/g for biosilica-modified silver ions. The average pore diameter of the materials varies from 2.97 nm to 4.40 nm, while the total pore volume reaches over 0.25 cm^3^/g. As can be seen from Table 1, the specific surface area, BET, of hierarchical materials is larger than that of the starting commercial zeolite, which is not surprising if one considers the dispersion of larger particles. A large volume of the formed mesopores (a remarkable increase from 0.03 to 0.30 cm^3^/g) is characteristic of the synthesized micro-mesoporous zeolites. The average pore diameter varies from 2.46 nm to 5.84 nm. There is a decrease in the specific micropore volume of the synthesized samples in relation to that of the commercial zeolites, which is due to the mixed composition of the hybrid samples (zeolite with mesoporous silica).

Transmission electron microscopy (TEM) images of the hierarchical zeolites are presented in Figure S1 in Supplementary Materials. The TEM images reveal the presence of metal nanoparticles (ruthenium or silver) on the surface of the materials. The oval-shaped metal nanoparticles, 2–100 nm in size, are gathered in clusters of irregular shapes.

### 2.2. Photocatalytic Removal of Bisphenol A

Photocatalytic removal of bisphenol A under visible light is difficult, mainly for the two following reasons. The first is that bisphenol A absorbs UV light but does not absorb visible light, and photoremoval by visible light is inefficient compared to that upon UV irradiation, while the second reason is that bisphenol A is present in the environment in low concentrations, hence, it’s almost complete photoremoval requires effective catalysts. In designing a suitable photocatalyst, it should be taken into account that such a catalyst should efficiently capture visible light and generate electron-hole pairs for the chemical reactions. Commonly used photocatalysts include semiconductor particles and, in selected cases, plasmonic particles. However, the recombination of electron-hole pairs is rapid, as their lifetimes are on the picosecond and femtosecond scales for the semiconductor and plasmonic nanoparticles, respectively [3,32,33,34,35,36]. Therefore, photocatalytic reactions are generally very inefficient, as has been observed for different types of photocatalysts used for bisphenol A removal [37,38,39,40,41,42]. In this regard, the proposed photoremoval based on the use of hierarchical zeolites and diatom biosilica has the advantage of offering near-complete removal of bisphenol A into smaller fragments under visible-light irradiation, and the catalyst can be easily prepared on the milligram to gram scale with reusability.

To test the photocatalytic performance, hierarchical zeolites and diatom biosilica modified with ruthenium ions and silver were added to the bisphenol A solution for photocatalytic removal reaction. The results of the reaction are shown in Table 2. In the first 30 min of the reaction in the dark, all samples were in the adsorption stage. After this time, the adsorption equilibrium state was reached. In the reaction taking place for 30 min in the dark, it was observed that about 30% of BPA was clearly adsorbed in the pH 5–7 range, while at pH 11, the adsorption was greater, reaching a value of about 57%. After the onset of irradiation, no significant photoremoval was observed at pH 5 and pH 11, while at pH 7, the removal percentage was about 90%.

According to the results obtained, the best photocatalyst turned out to be biosilica modified with ruthenium ions. The highest degree of bisphenol A removal, reaching as much as 99.6% was obtained using blue light (Figure S2 in Supplementary Materials). High bisphenol A removal values of 70.5% and 62.4% were obtained using green and cyan light, respectively. The second-best photocatalyst turned out to be biosilica modified with silver ions. In the presence of this photocatalyst, the highest degree of bisphenol A removal was recorded using green, red, and yellow lights. This may be due to the fact that silica frustules extracted from diatom cultures exhibit high photoluminescence activity associated with light emission in the middle ultraviolet (290–300 nm) under UV radiation, emission (493 nm), and excitation (480 nm) in the narrow blue region, and emission in the narrow blue region and emission in the green region (498–525 nm) of the visible spectrum under UV radiation [14,20].

In contrast, the samples containing hierarchical zeolites as photocatalysts yielded low values of bisphenol A removal. UV-Vis spectra have been added as additional material (Figures S3–S6 in Supplementary Materials). The photocatalytic behavior of two pure carriers (FAU and bio-SiO_2_) without metals is characterized in Figures S7 and S8 in Supplementary Materials. Bisphenol A removal in the presence of these carriers reaches a maximum of about 25%. For the sake of comparison, the photoremoval of bisphenol A was carried out without the use of a catalyst. Bisphenol A removal was below 5% after 270 min of running the process (blank tests, Figure S9). Leaching tests were carried out in order to check whether the active species (Ru or Ag) were leached into the solution during the photoremoval process. The metal ion content of the solution was less than 5% (Supplementary Materials, Figures S10–S13).

The results presented here are the first of their kind. The studies with the use of these materials in the photoremoval of bisphenol A are continued and will make the basis of a separate paper.

#### 2.2.1. Effect of Temperature and pH on the Photocatalytic Removal of Bisphenol A for Ruthenium Ion-Modified Diatom Biosilica

**Figure 4:**
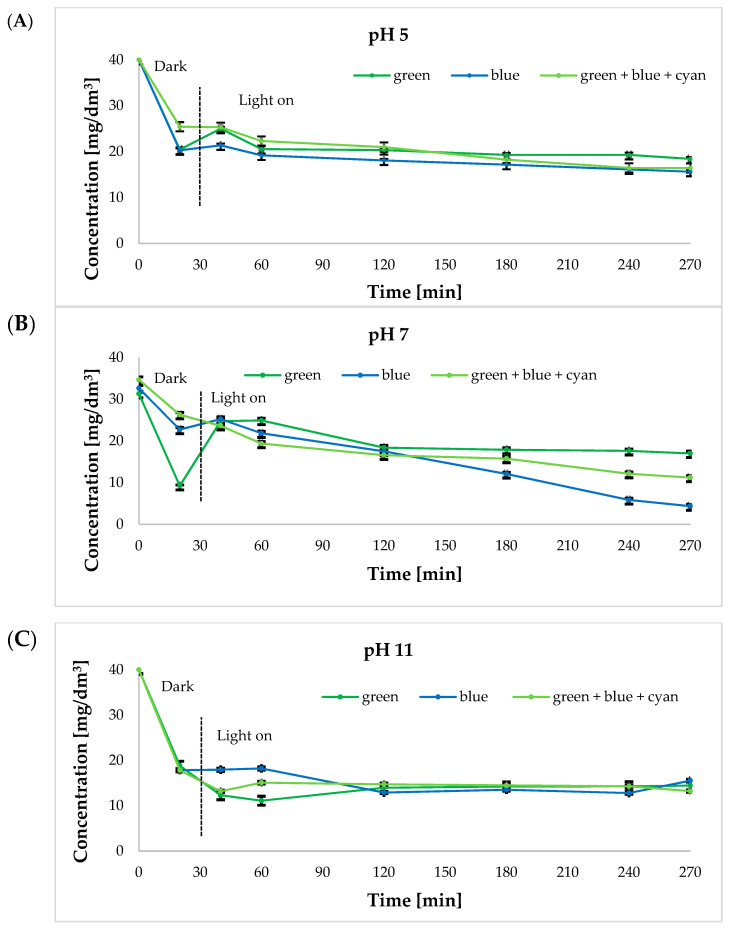
Time dependence of absorbance for 40 mg/dm^3^ bisphenol A solutions under green, blue, and green+blue+cyan under light irradiation at 25 °C at different solution pH: (**A**) pH 5; (**B**) pH 7; (**C**) pH 11 in the presence of ruthenium ion-modified biosilica.

**Figure 5:**
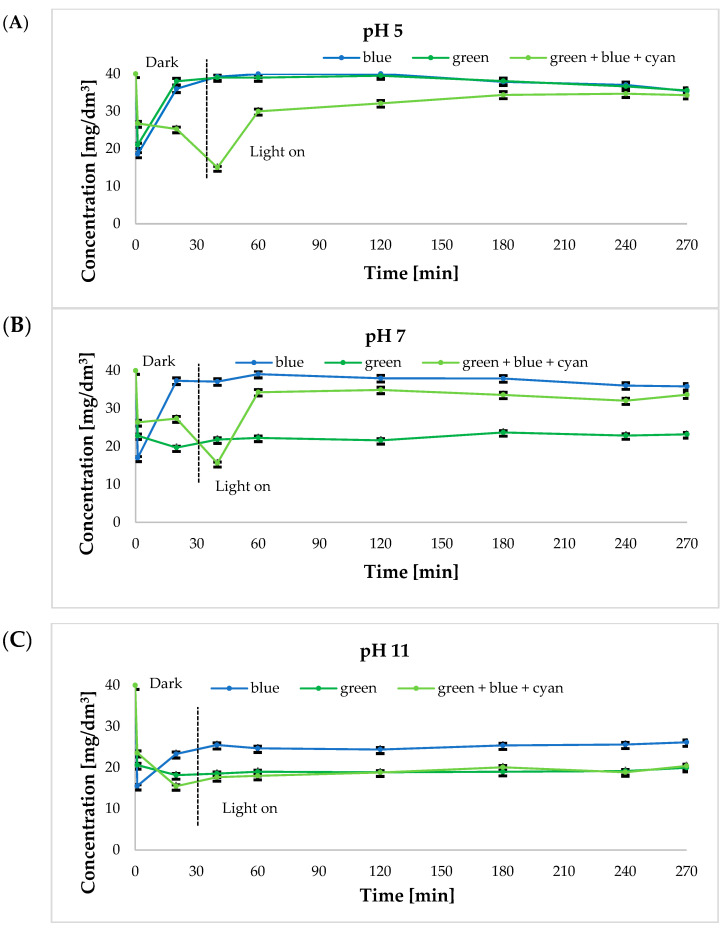
Time dependence of absorbance for 40 mg/dm^3^ bisphenol A solutions under green, blue, and green+blue+cyan light irradiation at 65 °C at different solution pH values using ruthenium ion-modified biosilica; (**A**) pH 5; (**B**) pH 7; (**C**) pH 11.

Based on the results collected in Table 1, both the most effective catalyst and the three colors of visible light, at which the highest degree of bisphenol A removal was obtained, were selected. Figure 4 and Figure 5 show graphs of the time dependence of absorbance for 40 mg/dm^3^ bisphenol A solutions (absorbance: 0.731 a.u.) under green, blue, and green+blue+cyan light irradiation at 25 °C and 65 °C at different solution pH values. Indeed, pH is an important factor in bisphenol removal [11]. Depending on the nature of organic pollutants, an increase in pH has a positive or negative effect on the rate of their removal and consequently affects the rate of mineralization of the solution. Under various conditions, Tao and colleagues have studied the effect of pH on BPA removal in the pH range of 2–12 using Ti-MCM-41 material [43]. BPA removal efficiency increased with pH values up to 8, above which the removal efficiency began to decrease, suggesting an optimum pH value of around 8 for the best performance. In turn, the pH value in the starting solution affects the formation of hydroxyl radicals. In addition, pH affects the ionization of reactants and products. The molecules of BPA are neutral in acidic environments and become negatively charged under alkaline conditions. It is also suggested that in an alkaline solution, OH^−^ radicals are more easily generated by oxidizing more available hydroxide ions. In order to determine the optimal pH for the photocatalytic removal of BPA using hierarchical zeolites and diatom biosilica modified with ruthenium ions and silver, three different solutions were prepared whose pH was maintained at 5, 7, and 11, respectively.

The initial concentration of BPA was 40 mg/dm^3^.

It was observed that the degree of BPA disappearance was strong under neutral and alkaline pH conditions (Figure 4 and Figure 5). A possible explanation of a fast BPA disappearance at pH 7 (Figure 4B) and pH 11 (Figure 4C) is the amphoteric behavior of the semiconductor material and the change in the surface charge properties of the photocatalyst [11].

For the bisphenol A removal reaction carried out at 25 °C (Figure 4A) at pH 5, the solution was noted to discolor from its initial light green with increased time of exposure to light. At pH 7, on the other hand, the color of the solution changed from light green to turquoise. At pH 11, a steady foaming of the solution was noted with the increased time of the sample exposure to light. A color change from dark green/black to light green was also observed (Figure S14 in Supplementary Files).

The obtained absorbance values for bisphenol A solutions at 65 °C (Figure 5) were compared to the results obtained when the process was carried out at a lower temperature of 25 °C. The most favorable results of absorbance measurements were obtained for the green light and the sum of the green, blue, and cyan lights. All results obtained for individual light colors, including blue light, were similar to each other. As for the effect of the pH value on the photocatalytic removal process, the lowest absorbance values were obtained for bisphenol A upon illumination of the solutions of pH 11 (Figure 5C).

Based on the data shown in Figure 4 and Figure 5, bisphenol A removal was observed after about 40 min of running the reaction (green light and blue light), followed by a sharp increase in its concentration. We found that this is due to the initial adsorption of bisphenol A on the surface of the catalyst used and its subsequent desorption from the surface of the material. Since pH mostly influences adsorption/desorption equilibria, this affects the overall removal percentage. The removal of bisphenol A is the result of both degradation and adsorption processes. When the adsorption process is prevalent, the pH dependency is higher. This is confirmed by the low removal rate observed at 65 °C, where desorption is promoted (after initial strong adsorption). Adsorption/desorption phenomena occur throughout the entire period of the experiment, both in the dark, with the major contribution to bisphenol A removal, as well as under light irradiation where only bisphenol A molecules closer to the ruthenium photoactive sites would be degraded [44].

#### 2.2.2. Qualitative Analysis of Bisphenol A removal Products by ESI/HPLC-MS Method

**Figure 6:**
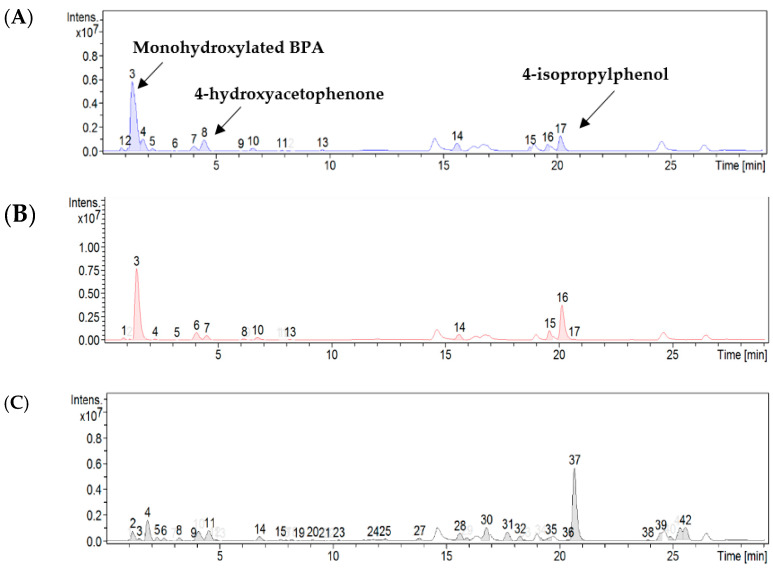
Chromatogram of bisphenol A removal products using biosilica modified ruthenium ion: (**A**) pH 5, T = 65 °C; (**B**) pH 7, T = 65 °C; (**C**) pH 11, T = 65 °C.

**Figure 7:**
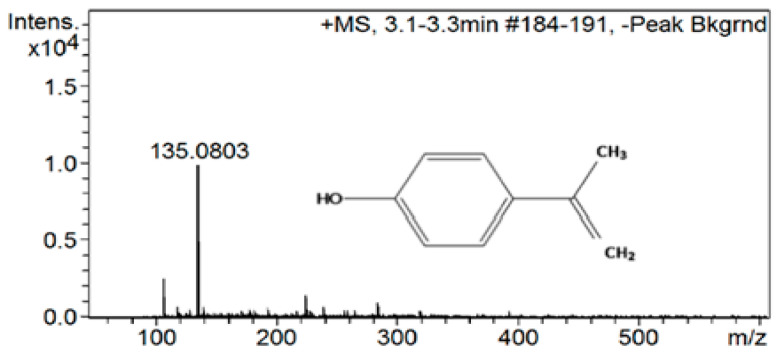
ESI-MS(+) for 4-isopropylphenol with molecular formula C_9_H_11_O ([M + H]^+^, *m/z*—135.0803), mass range 50–600 *m/z*.

High-performance liquid chromatography (HPLC), coupled with mass spectrometry with ionization by electrodispersion in an electric field ESI (ionization in the positive ion mode), was applied for the qualitative determination of products of photocatalytic removal of bisphenol A. This method was applied to the material most efficient from among those studied, i.e., ruthenium ion-modified biosilica. Figure 6A–C show chromatograms of bisphenol A removal products obtained using ruthenium ion-modified biosilica in three different reaction environments, i.e., at pH 5, 7, and 11. The following compounds were identified as the main reaction products: monohydroxylated BPA ([M + H]^+^, *m/z*—245.2533); 4-hydroxy acetophenone ([M + H]^+^, *m/z*—137.1457), and 4-isopropyl phenol ([M + H]^+^, *m/z*—135.0803) [36]. An exemplary ESI-MS(+) mass spectrum for 4-isopropylphenol with molecular formula C_9_H_11_O ([M + H]^+^, *m/z*—135.0803), mass range 50–600 *m/z* is shown in Figure 7.

**Figure 8:**
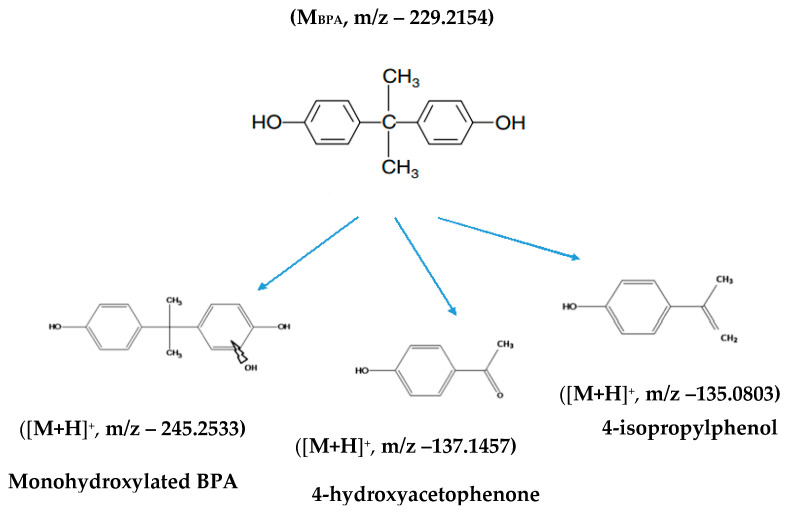
The decomposition products of bisphenol A.

The decomposition products of bisphenol A are shown in Figure 8.

## 3. Materials and Methods

### 3.1. Synthesis of Hierarchical Zeolites Based on FAU-Type Commercial Zeolite Modified with Silver Ions

The synthesis of the aforementioned materials was carried out in two stages (Figure S15 in Supplementary Materials):

#### 3.1.1. STAGE I

The preparation of a hierarchical material based on commercial FAU-type zeolite was based on dispersing commercial FAU zeolite (Alfa Aesar, Haverhill, MA, USA) in an amount of 0.50 g in a mixture containing 100.00 g distilled water, 1.25 g ammonia (StanLab, Lublin, Poland), 60.00 g of ethanol (StanLab, 96%), and 0.35 g of CTABr (cetyltrimethylammonium bromide) (Fluka Analytical). The whole mixture was placed in a polyethylene bottle and ultrasonicated for a period of 30 min at 65 °C. After this time, 0.56 g of tetraethyl orthosilicate (TEOS, silicon source) (Aldrich Chemistry, Saint Louis, MI, USA) was added to the solution as a source of silicon. In a further step of the synthesis, the whole mixture was stirred on a magnetic stirrer for 4 h at 65 °C, after which the resulting precipitate was drained off on a glass funnel using a filter strainer, washed with a mixture of distilled water—ethyl alcohol—in a ratio of 1:1, and left to dry in the air at room temperature. After drying, the material was calcined for 5 h at 550 °C.

TEOS molecules are incorporated into the interblock space and undergo simultaneous hydrolysis to form stable SiO_2_ filaments between the individual sheets and the surfactants (CTABr), used for intercalation or swelling [45].

#### 3.1.2. STAGE II

The material obtained in Stage I was subjected to wetting impregnation with silver ions using silver nitrate (Aldrich). A mixture containing 50.0 cm^3^ of demineralized water (Honeywell, Charlotte, NC, USA) and silver(I) nitrate (3% by weight of the carrier; Alfa Aesar, 99.9%) was added to the carrier (0.5 g) and treated with an ultrasound for 3 h. Then, the whole mixture was subjected to stirring for 24 h at 25 °C. In the next step of the synthesis, the catalysts were dried at 60 °C to evaporate the solvent.

### 3.2. Synthesis of Hierarchical Zeolites Based on FAU-Type Commercial Zeolite Modified with Ruthenium Ions

The preparation of hierarchical material based on commercial FAU-type zeolite was based on dispersing the commercial FAU zeolite (Alfa Aesar) in the amount of 0.50 g in a mixture containing 100.00 g distilled water, 1.25 g ammonia (StanLab), 60.00 g ethanol (StanLab, 96%) and 0.35 g CTABr (cetyltrimethylammonium bromide) (Fluka Analytical). The whole mixture was placed in a polyethylene bottle and ultrasonicated for a period of 30 min at 65 °C. After this time, 0.56 g of tetraethyl orthosilicate (TEOS) (Aldrich Chemistry) was added to the solution as a silicon source, followed by 0.0122 g of ruthenium source, which was chloropentaaminoruthenium(III) chloride (Sigma Aldrich, >98%). In a further step of the synthesis, the whole mixture was stirred on a magnetic stirrer for 24 h at 65 °C, after which the resulting precipitate was drained on a glass funnel using a filter strainer, washed with a mixture of distilled water-ethyl alcohol at a ratio of 1:1, and allowed to air-dry at room temperature. After drying, the material was calcined for 5 h at 550 °C (Figure S16 in Supplementary Materials).

### 3.3. Synthesis of Biosilica Modified with Silver Ions or Ruthenium Ions

Synthesis of diatom biosilica doped with silver(I) nitrate was carried out by dispersing diatom biosilica obtained from *Pseudostaurosira trainorii* strain (0.5 g) in an ultrasonic bath for 3 h in a mixture containing 50.0 cm^3^ of demineralized water (Honeywell) and silver(I) nitrate (3% by weight relative to the carrier; Alfa Aesar, 99.9%) or methanol (Honeywall) and pentaaminoruthenium(III) chloride (3% by weight relative to the carrier). The mixture was then stirred for 24 h at 25 °C. In the next step, the catalysts were dried at 60 °C to evaporate the solvent (Figure S17 in Supplementary Materials).

### 3.4. Labelling of the Materials Studied

Henceforth the materials studied will be labeled as follows:
**Zeolite FAU_CTABr_3%AgNO_3_**Hierarchical zeolite based on FAU-type commercial zeolite modified with 3 wt. % AgNO_3_ silver nitrate solution**Zeolite FAU_CTABr_Ru**Hierarchical zeolite based on FAU-type commercial zeolite modified with ruthenium**Ag Bio**Biosilica modified with silver ions**Ru Bio**Biosilica modified with ruthenium ions

### 3.5. Characterization of the Materials

Preliminary characterization of the materials was performed using X-ray diffraction (XRD) and low-temperature nitrogen adsorption/desorption isotherms.

X-ray diffraction studies were performed on a Bruker AXS D8 Advance diffractometer with a Johannson monochromator and a LynxEye strip detector. The CuK_α_ radiation source generated a wavelength of λ = 0.154 nm in the low-angle range of 2θ = 0.6–8.0° (with an accuracy of 0.02°) and the high-angle range (with an accuracy of 0.05°) of 2θ = 6.0–60.0°.

Measurements of low-temperature nitrogen adsorption/desorption isotherms were made using a Quantachrome Autosorb iQ instrument. Before performing the actual measurement, the test samples were degassed under a vacuum at 110 °C for 24 h. Isotherms were recorded at ca. −196 °C, in the relative pressure range p/p_0_ from 0.02 to 1.00.

The morphology and structure of the obtained materials were examined by transmission electron microscopy (TEM, JEOL-2000).

### 3.6. Photoremoval of Bisphenol A

An appropriate amount of the catalyst (0.003 g) was weighed and placed in a vial to which 5 cm^3^ of an aqueous bisphenol A solution of 40 mg/dm^3^ (pH 7) was successively added. The vial containing the mixture, provided with a magnetic dipole, was placed on a magnetic stirrer and sealed in a reactor, where adsorption equilibrium was achieved in the dark upon magnetic stirring for 30 min. The mixture was then irradiated using a specific wavelength (see Table 3). This irradiation was carried out for a period of 4.5 h, with absorbance measurements made after 20, 40, 60, 120, 180, 240, and 270 min of illumination. The obtained results were presented as graphs for each color of visible light.

The electromagnetic wavelengths used are given in Table 3. Absorbance measurements were made separately for each sample. Emission spectra of monochromatic light sources were obtained from the LED distributor and are presented in the Supplementary Materials (Figure S18).

For a standard solution of bisphenol A with a concentration of 40 mg/dm^3^ and a pH of 6.8, the characteristic wavelength was 283 nm [absorbance value was 0.731 a.u.].

Based on a detailed analysis of the absorbance measurements of bisphenol A solutions (wavelength 283 nm), the most effective catalyst and three colors of visible light were selected for which the highest degree of bisphenol A removal was obtained. The chosen catalyst was ruthenium-modified biosilica, while the visible light colors were green (525 nm), blue (450 nm), and cyan (500 nm). Subsequent photoremoval reactions of bisphenol A were then carried out using ruthenium-modified biosilica as a catalyst for the aforementioned reactions. For this purpose, solutions of bisphenol A (concentration 40 mg/dm^3^) of pH 5 and pH 11 were prepared in 250 cm^3^ flat-bottomed flasks. The process was carried out at two preset temperatures: 25 °C and 65 °C. The percentage of BPA removal was calculated using Equation (1), where *C*_1_ is the initial concentration of BPA and *C* is the concentration of bisphenol A over time. The percentage photoremoval of BPA was calculated from the equation:

(1) $$\%\mathrm{photoremoval}\;\mathrm{of}\;\mathrm{BPA}=\frac{C1-C}{C1}\times 100$$

Equation (1): The percentage of BPA removal.

### 3.7. Blank Tests and Leaching Tests

Leaching tests were carried out for all reactions tested under the general reaction conditions used in photocatalytic studies (as described above). The solid catalyst was removed from the reaction mixture after 3 h by centrifugation, followed by filtration using a plastic filter syringe. Bisphenol A was added to the filtrate (in the amount corresponding to a concentration of 40 mg/dm^3^). The filtrate was then used to carry out the photocatalytic reaction.

Blank experiments were carried out for all reactions tested, using only bisphenol A solution, under identical test conditions as for the catalyzed reactions.

### 3.8. Analysis of Bisphenol A Removal Products Using ESI/HPLC-MS

Bisphenol A removal products were analyzed using a QTOF mass spectrometer (Impact HD, Bruker Daltonics, Billerica, MA, USA) in positive ion mode and an Ultimate 3000 liquid chromatograph (Thermo Scientific/Dionex, Waltham, MA, USA). Method parameters are shown below and in Table 4:

Kinetex 2.6 um C18 column (100 × 2.10 mm)

Column temperaturę: 35 °C

Flow rate of mobile phase 0.3 cm^3^/min

Phase A: H_2_0 + 0.1% FA

Phase B: ACN + 0.1% FA

Injection volume 10.0 µL

Scanning range *m/z* = 50–600

Positive mode.

## 4. Conclusions

Based on the performed studies, it was found that the hierarchical zeolites and diatom biosilica, modified with silver and ruthenium ions, used for the first time as photocatalysts for bisphenol A removal, proved to be effective. In addition, it was proved that:

- the efficiency of bisphenol A removal was significantly influenced by the modification of the obtained photocatalysts with ruthenium or silver ions, for the materials modified with ruthenium ions, an increase in bisphenol A removal was noted,
- the degree of bisphenol A removal was dependent on the electromagnetic wavelength used, it was the highest at the wavelengths corresponding to blue, green, and cyan light,
- the optimal temperature favorably affecting the removal of bisphenol A is 25 °C. As for the effect of pH values on the photocatalytic removal process, the best results were recorded for the samples whose pH values were 7 and 11,
- the best photocatalyst is ruthenium ion-modified biosilica, in whose presence up to 99% removal of bisphenol A was achieved, the highest degree of bisphenol A d removal was obtained at electromagnetic wavelengths ranging from 450 nm to 525 nm;
- the products of photocatalytic removal of bisphenol A are: 4-hydroxy acetophenone, 4-isopropyl phenol, and monohydroxylated bisphenol A.

## Figures and Tables

**Table 1:** Porous structure parameters of the materials studied.

Sample	Specific Surface Area [m^2^/g]	Pore Volume [cm^3^/g]		Average Pore Diameter [nm]
		Total	Microporous volume	
Hierarchical zeolites				
**Commercial zeolite—FAU**	718	0.37	0.34	-
**Zeolite FAU_CTABr_3%AgNO_3_**	746	0.34	0.13	5.84
**Zeolite FAU_CTABr_Ru**	776	0.41	0.11	2.46
Biosilica				
**Biosilica**	30	0.43	-	3.93
**Ag Bio**	104	0.25	-	2.97
**Ru Bio**	39	0.27	-	4.40

**Table 2:** Summary of the results obtained in the photocatalytic removal of bisphenol A using selected catalysts (the time of light reaction—270 min, pH 7, T = 25 °C).

Light Color	Wavelength [nm]	Degree of Bisphenol A Removal [%]
**Zeolite FAU_CTABr_3%AgNO_3_**		
**Green**	525	**7.3**
**Blue**	450	**9.4**
**UV**	395–405	**8.3**
**Yellow**	595	**8.2**
**Cyan**	500	**16.1**
**Red**	620–630	**8.1**
**Green + Blue + Cyan**	450–525	**20.1**
**Zeolite FAU_CTABr_Ru**		
**Green**	525	**0.2**
**Blue**	450	**1.6**
**UV**	395–405	**2.2**
**Yellow**	595	**6.0**
**Cyan**	500	**35.7**
**Red**	620–630	**0.0**
**Green + Blue + Cyan**	450–525	**1.5**
**Ag Bio**		
**Green**	525	**28.7**
**Blue**	450	**8.3**
**UV**	395–405	**24.8**
**Yellow**	595	**37.2**
**Cyan**	500	**11.9**
**Red**	620–630	**31.6**
**Green + Blue + Cyan**	450–525	**15.1**
**Ru Bio**		
**Green**	525	**70.5**
**Blue**	450	**99.6**
**UV**	395–405	**41.5**
**Yellow**	595	**43.0**
**Cyan**	500	**62.4**
**Red**	620–630	**34.1**
**Green + Blue + Cyan**	450–525	**68.1**

**Table 3:** Wavelength values for selected colors of visible light.

**Light Color**	**Wavelength [nm]**
**UV**	395–405
**Blue**	450
**Cyan**	500
**Green**	525
**Yellow**	595
**Red**	620–630
**Green + Blue + Cyan**	450–525

**Table 4:** LC-MS method parameters.

Time [min]	A [%]	B [%]
0	90	10
1	90	10
30	30	70
32	30	70
34	90	10
39	90	10

## Data Availability

Not applicable.

## Supplementary Materials

The following supporting information can be downloaded at: https://www.mdpi.com/article/10.3390/ijms24032878/s1.
