# Supplementary material for: Photoremoval of Bisphenol A Using Hierarchical Zeolites and Diatom Biosilica
Source: Int J Mol Sci. 2023 Feb 2;24(3):2878. doi: 10.3390/ijms24032878 (PMC9918139; doi:10.3390/ijms24032878)
Supplement: Supplementary file 1 [file ijms-24-02878-s001.zip › ijms-2061357-supplementary.pdf]

SUPPLEMENTARY MATERIALS

Photoremoval of Bisphenol a Using Hierarchical Zeolites and Diatom Biosilica

Jagoda Chudzińska [1], Bartosz Woźniak [1], Myroslav Sprynskyy [2], Izabela Nowak [1] and Agnieszka Feliczak-Guzik [1,*]

[1] Faculty of Chemistry, Adam Mickiewicz University in Poznań, Uniwersytetu Poznańskiego 8, 61-614 Poznań, Poland;

[2] Faculty of Chemistry, Nicolaus Copernicus University in Toruń, 7 Gagarina Str., 87-100 Toruń, Poland

* Correspondence: agaguzik@amu.edu.pl; Tel.: +48-618-291-747

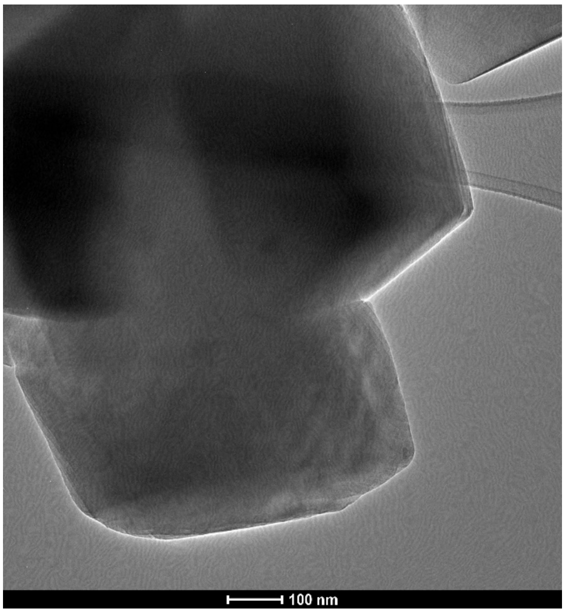

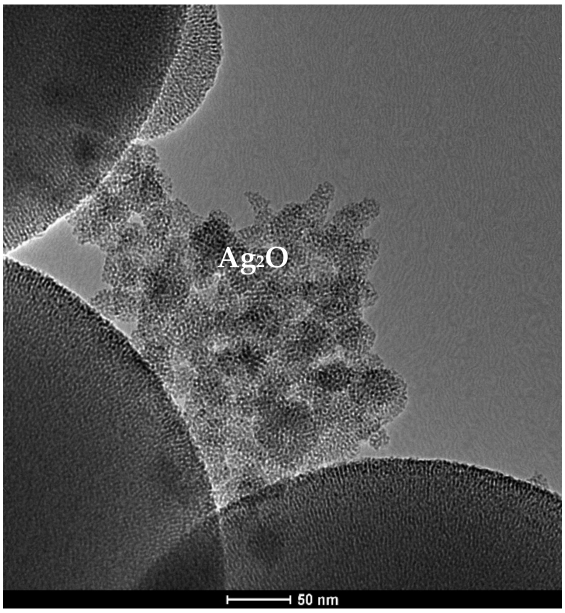

Commercial zeolite (FAU)

Zeolite FAU_CTABr_3%AgNO3

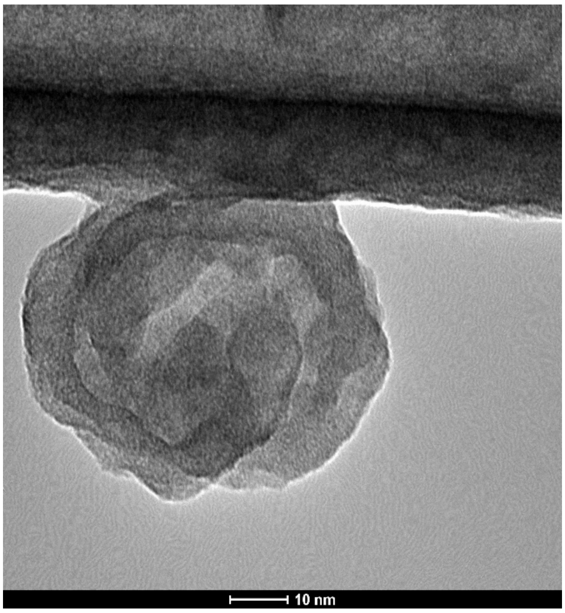

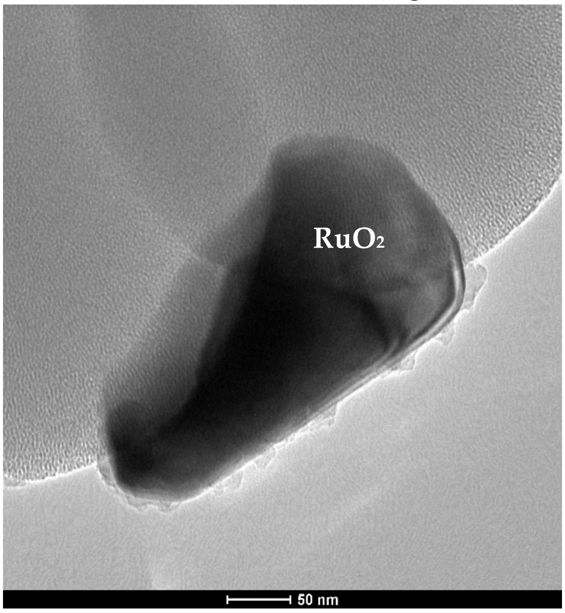

Zeolite FAU_CTABr_Ru

Figure S1. Transmission electron microscopy (TEM) images.

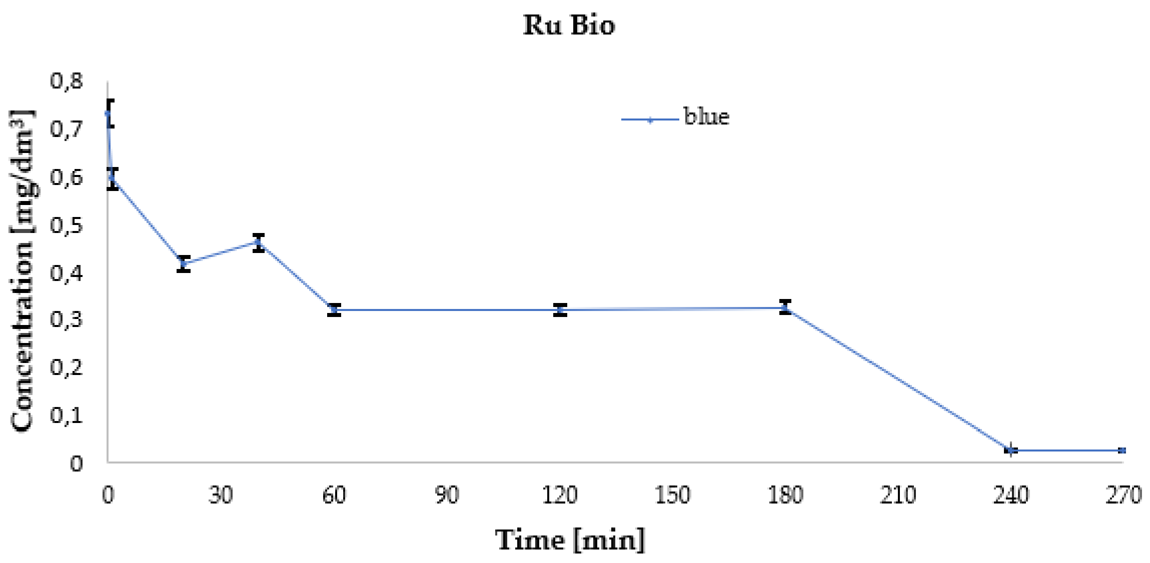

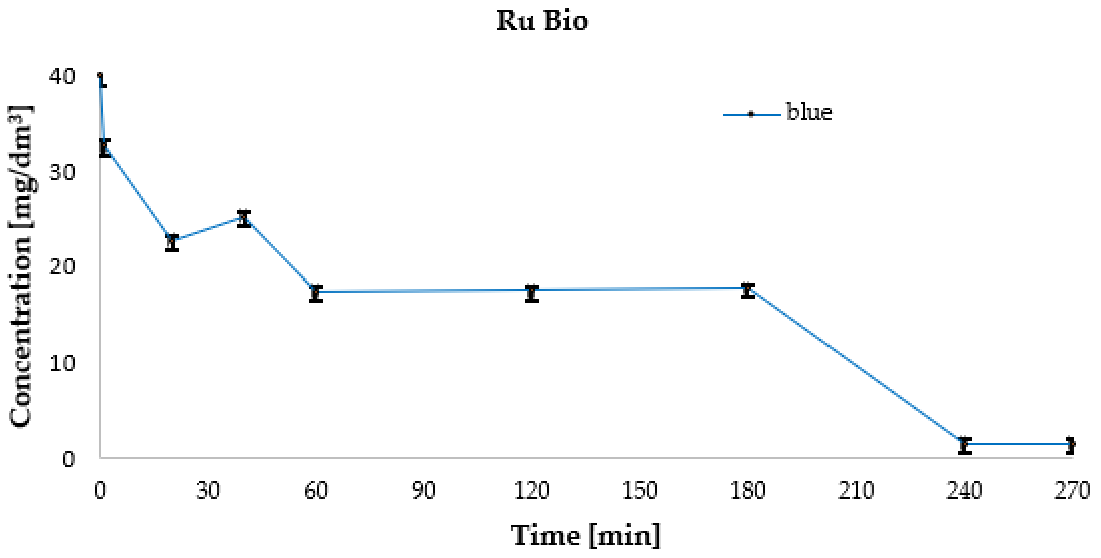

Figure S2. Time dependence of absorbance/concentration for 40 mg/dm^3 bisphenol A solutions under blue light irradiation at 25 °C, pH 7 in the presence of ruthenium ion- modified biosilica.

Photocatalytic removal of bisphenol A using a hierarchical zeolite based on FAU-type commercial zeolite modified with ruthenium ions

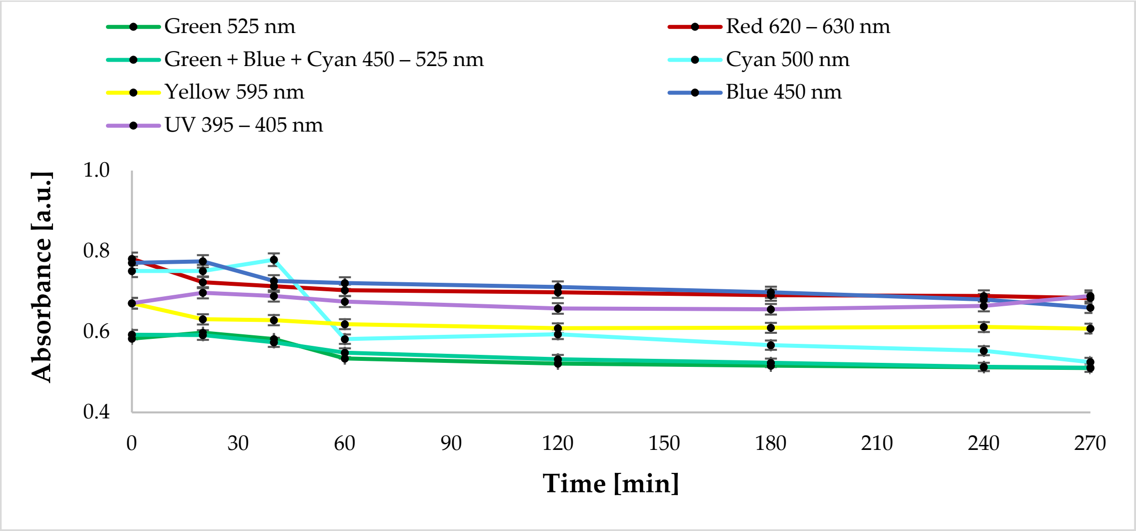

Figure S3. Time dependence of absorbance for 40 mg/dm^3 bisphenol A solutions under light irradiation at 25 °C, pH 7 using a hierarchical zeolite based on FAU-type commercial zeolite modified with ruthenium ions.

Photocatalytic removal of bisphenol A using a hierarchical zeolite based on FAU-type commercial zeolite modified with silver ions

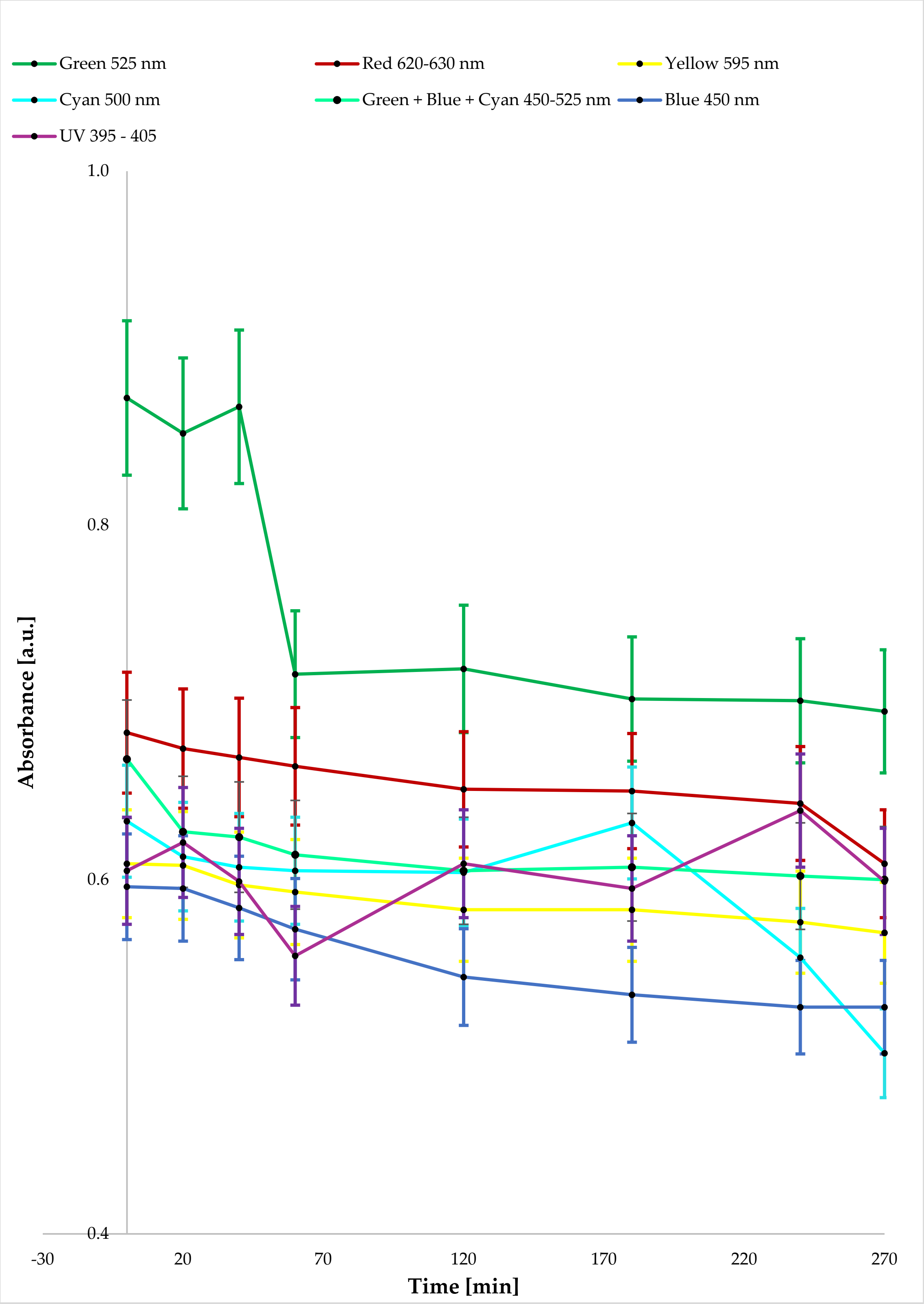

Figure S4. Time dependence of absorbance for 40 mg/dm^3 bisphenol A solutions under light irradiation at 25 °C, pH 7 using a hierarchical zeolite based on FAU-type commercial zeolite modified with silver ions.

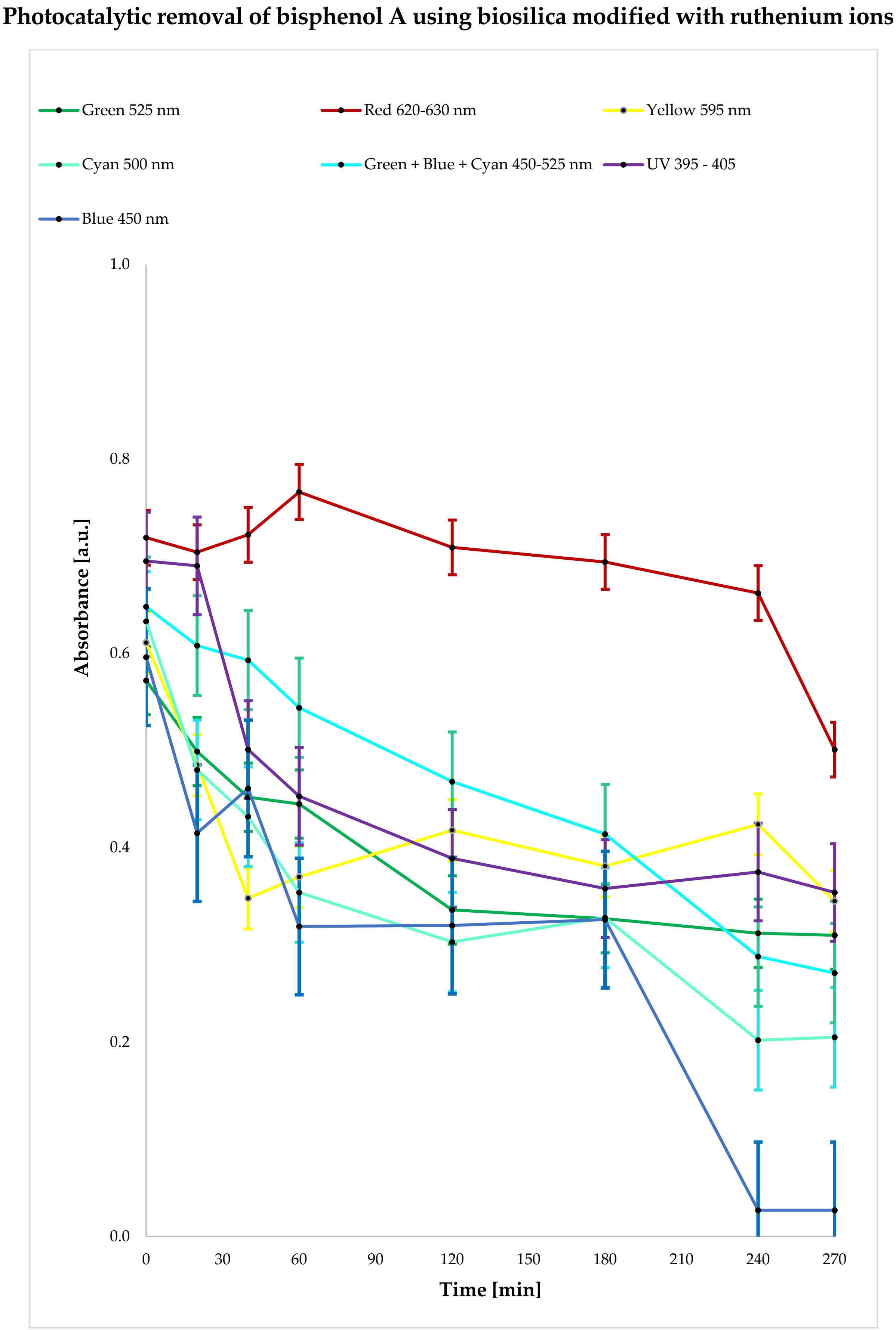

Figure S5. Time dependence of absorbance for 40 mg/dm³ bisphenol A solutions under light irradiation at 25 °C, pH 7 using biosilica modified with ruthenium ions.

Photocatalytic removal of bisphenol A using biosilica modified with silver ions

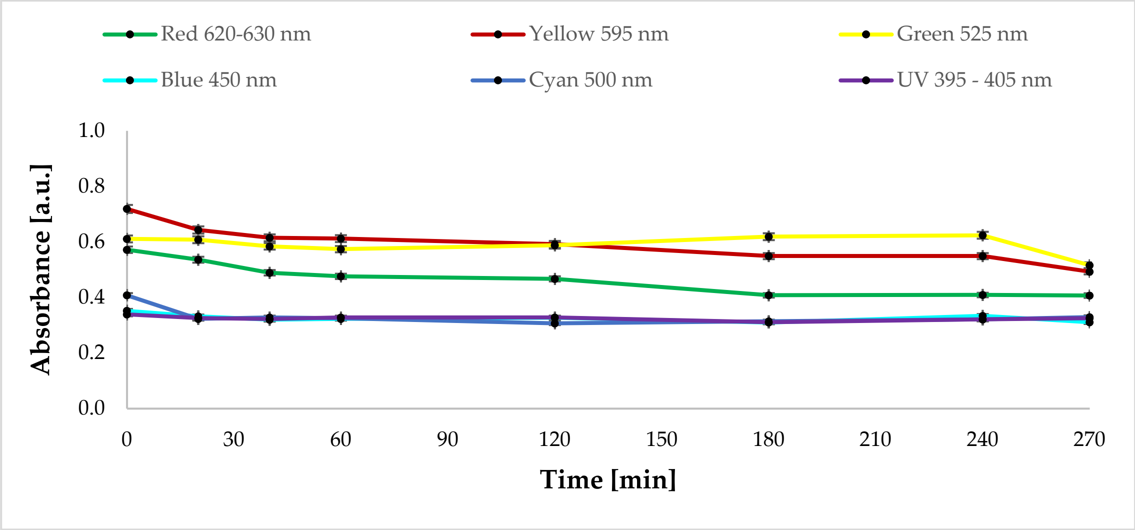

Figure S6. Time dependence of absorbance for 40 mg/dm^3 bisphenol A solutions under light irradiation at 25 °C, pH 7 using biosilica modified with silver ions.

Photocatalytic removal of bisphenol A using biosilica (bio-SiO_2)

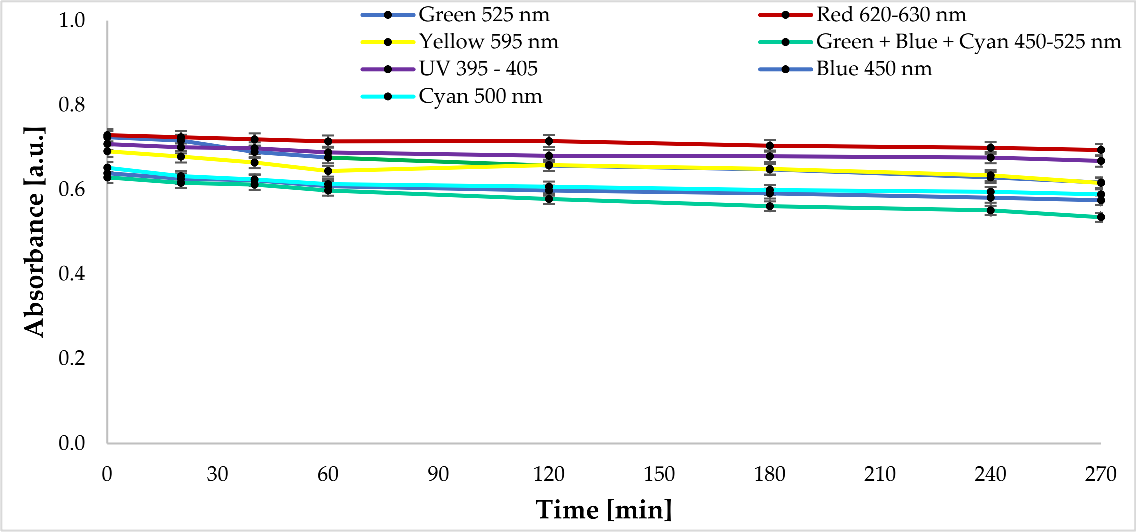

Figure S7. Time dependence of absorbance for 40 mg/dm^3 bisphenol A solutions under blue light irradiation at 25 °C, pH 7 using biosilica.

Photocatalytic removal of bisphenol A using commercial zeolite - FAU

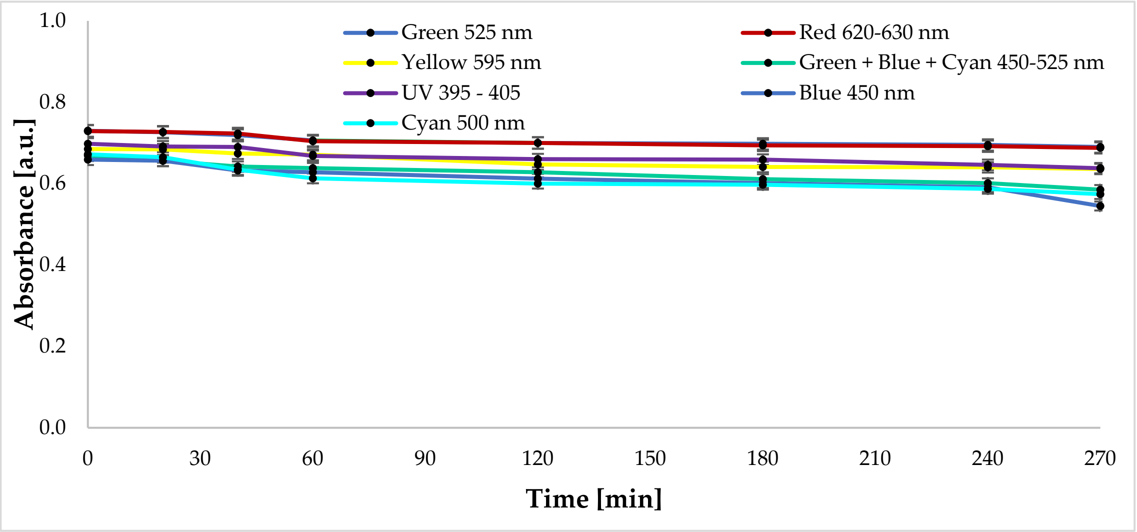

Figure S8. Time dependence of absorbance for 40 mg/dm^3 bisphenol A solutions under light irradiation at 25 °C, pH 7 using commercial zeolite – FAU.

Photocatalytic removal of bisphenol A without catalyst - blank

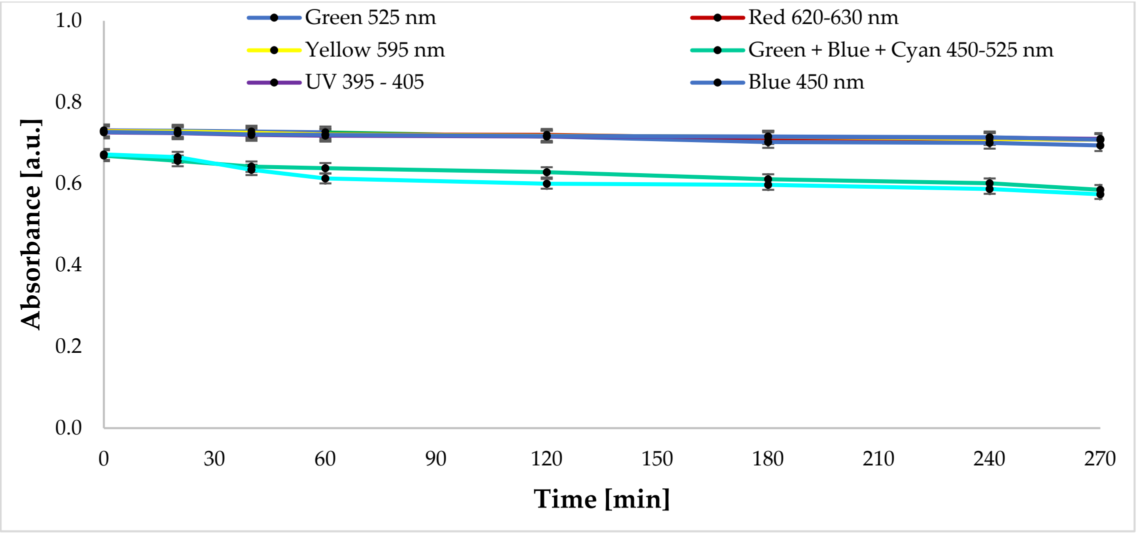

Figure S9. Time dependence of absorbance for 40 mg/dm^3 bisphenol A solutions under blue light irradiation at 25 °C, pH 7 without catalyst (blank).

Leaching tests

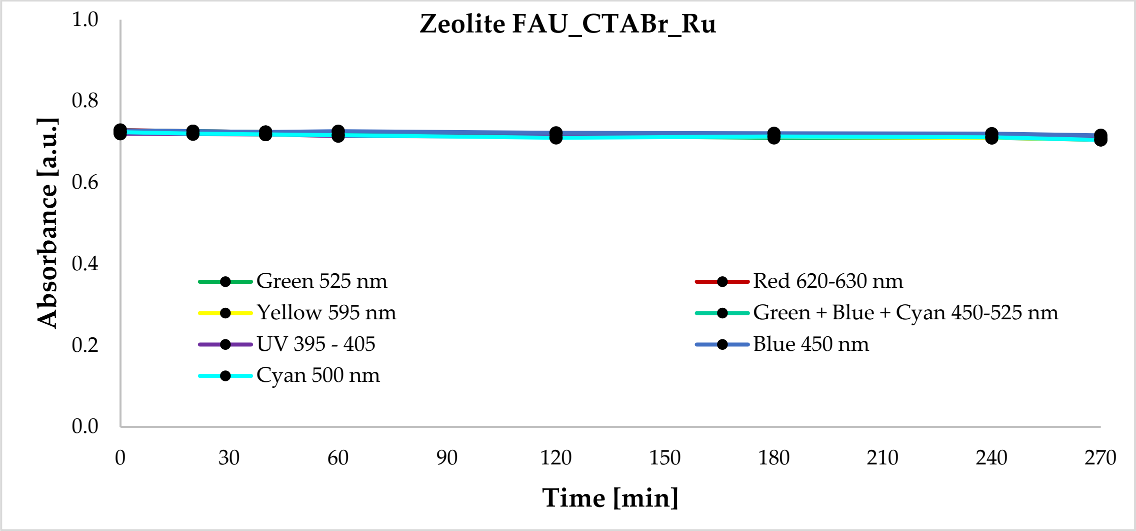

Figure S10. Leaching test – Zeolite FAU_CTABr_Ru; T = 25 °C; pH 7.

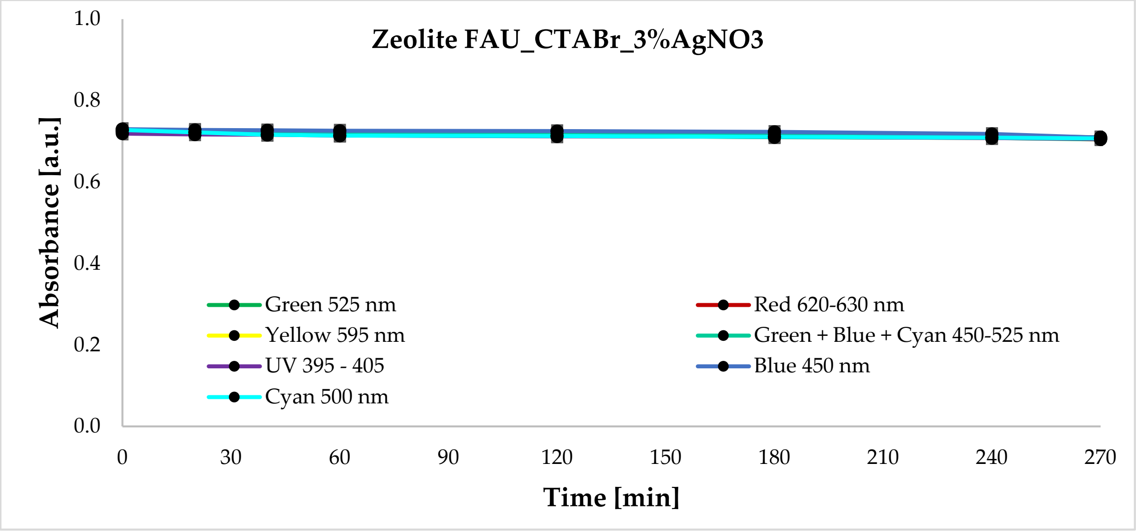

Figure S11. Leaching test – Zeolite FAU_CTABr_3%AgNO3; T = 25 °C; pH 7.

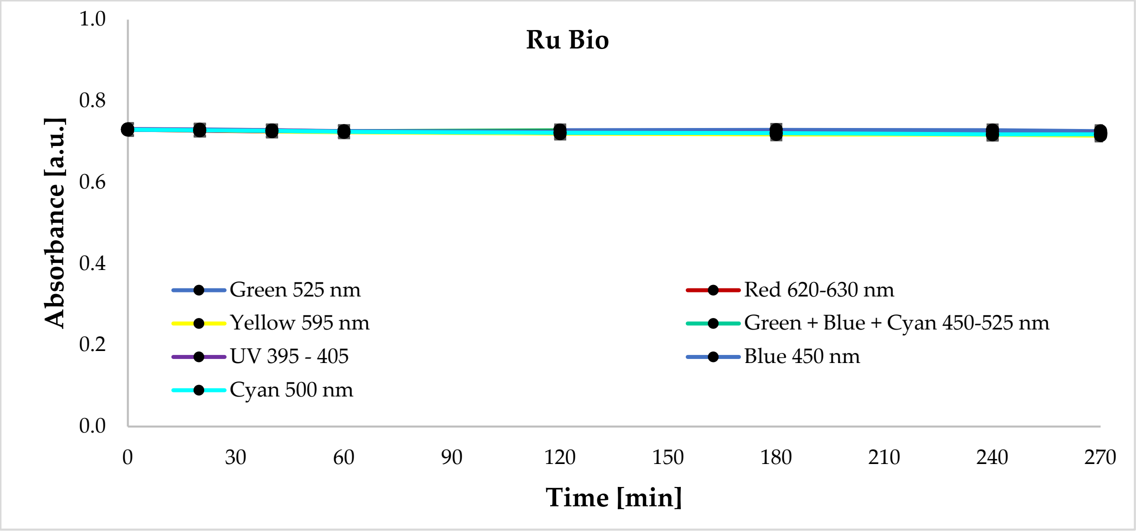

Figure S12. Leaching test – Ru Bio; T = 25 °C; pH 7.

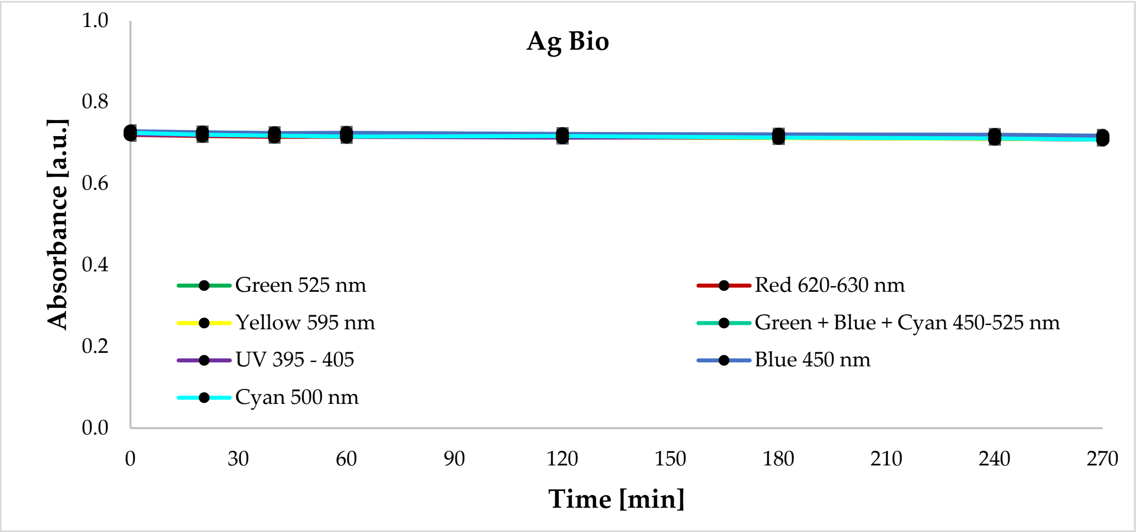

Figure S13. Leaching test – Ag Bio; T = 25 °C; pH 7.

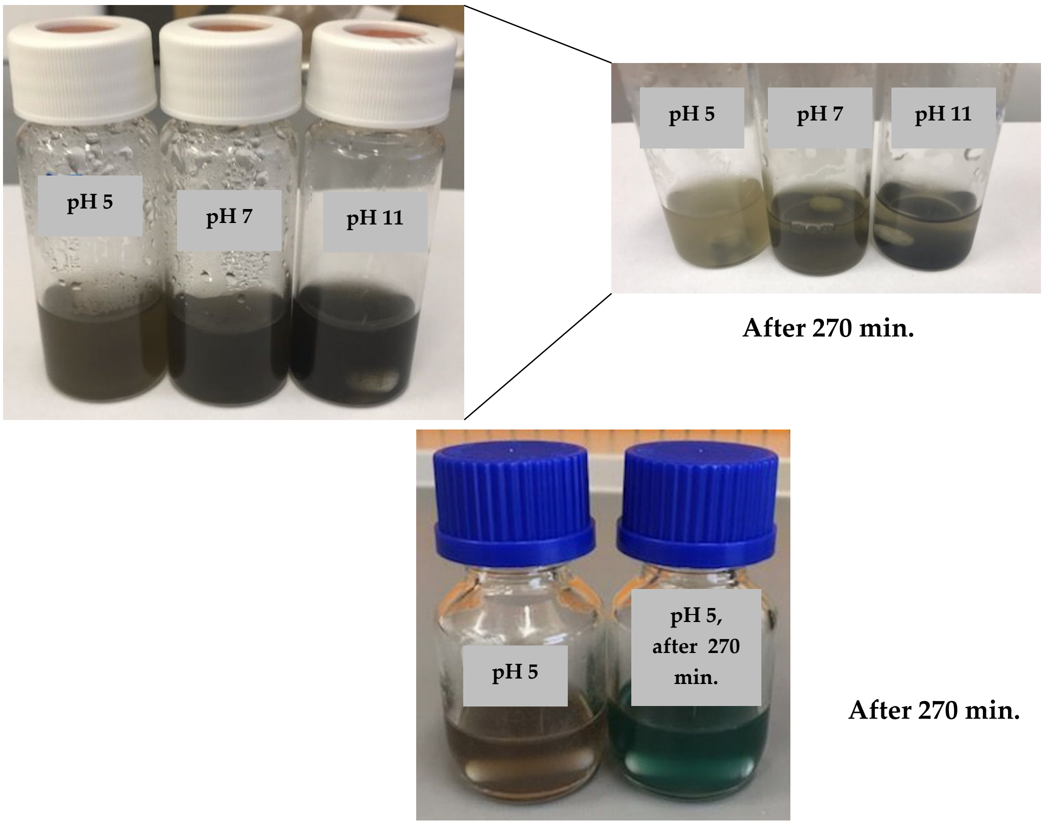

Figure S14. Effect pH on the photocatalytic **removal** of bisphenol A for ruthenium ion-modified diatom biosilica - Color change of bisphenol solution over time (T = 25 °C; green+blue+cyan light irradiation, at different solution pH values).

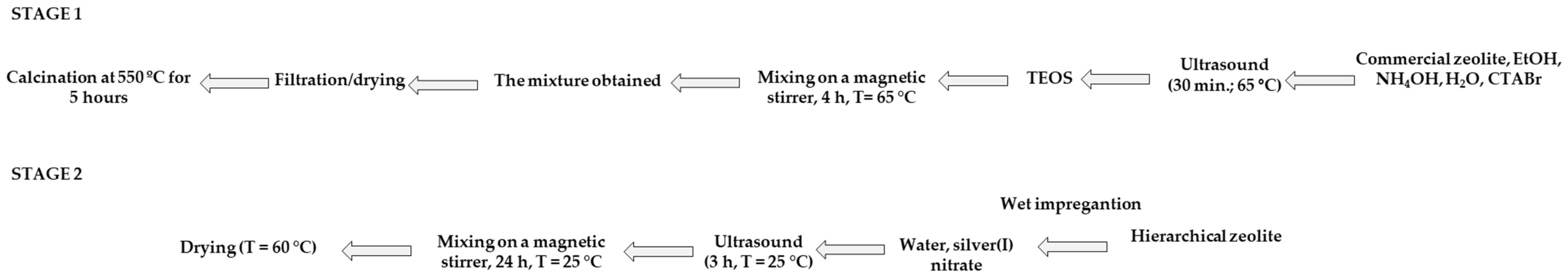

Figure S15. Synthesis of hierarchical zeolites based on FAU-type commercial zeolite modified with silver ions.

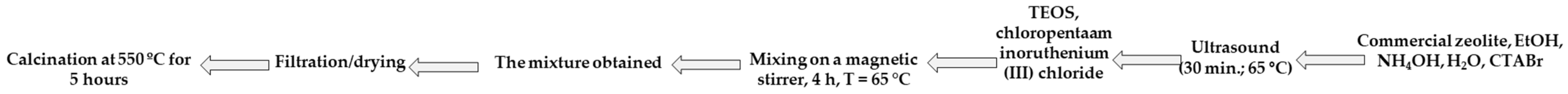

Figure S16. Synthesis of hierarchical zeolites based on FAU-type commercial zeolite modified with ruthenium ions.

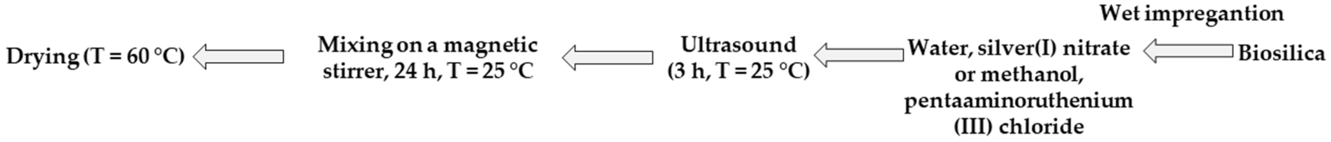

Figure S17. Synthesis of biosilica modified with silver ions or ruthenium ions.

Emission spectra of monochromatic light sources

Light color

UV
395 – 405 nm

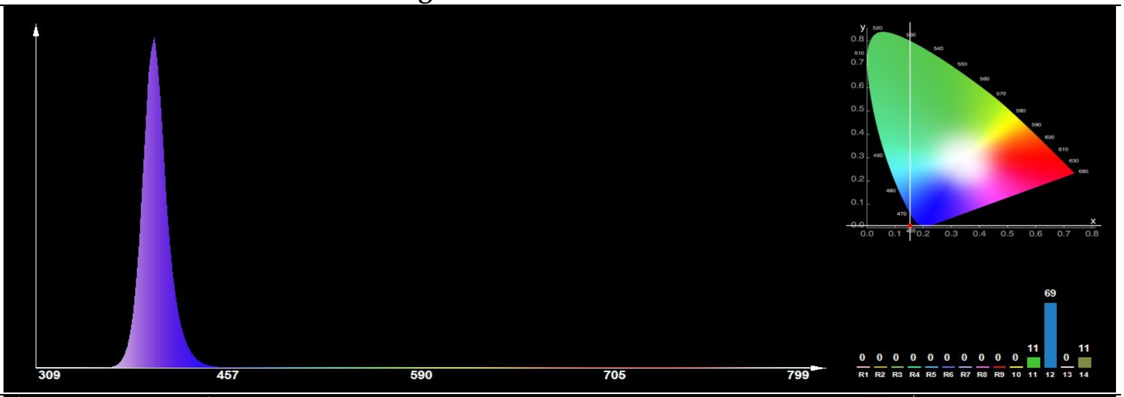

Blue
450 nm

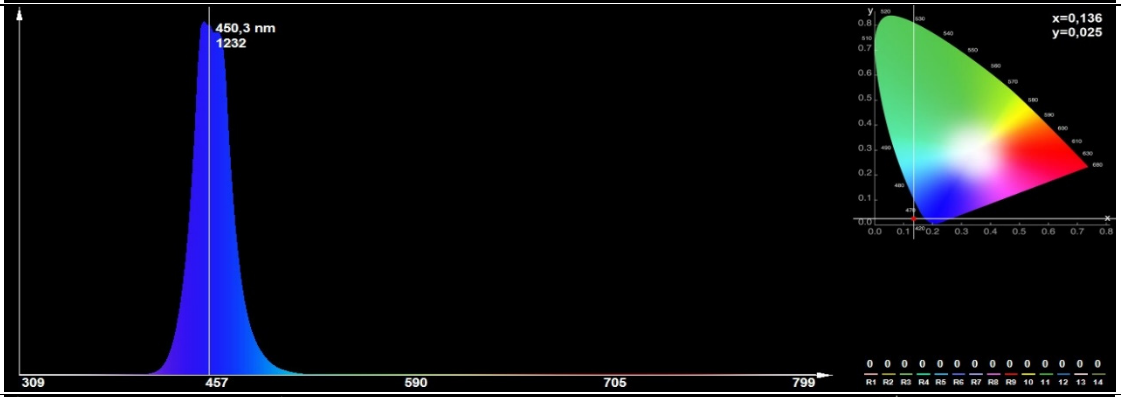

Cyan
500 nm

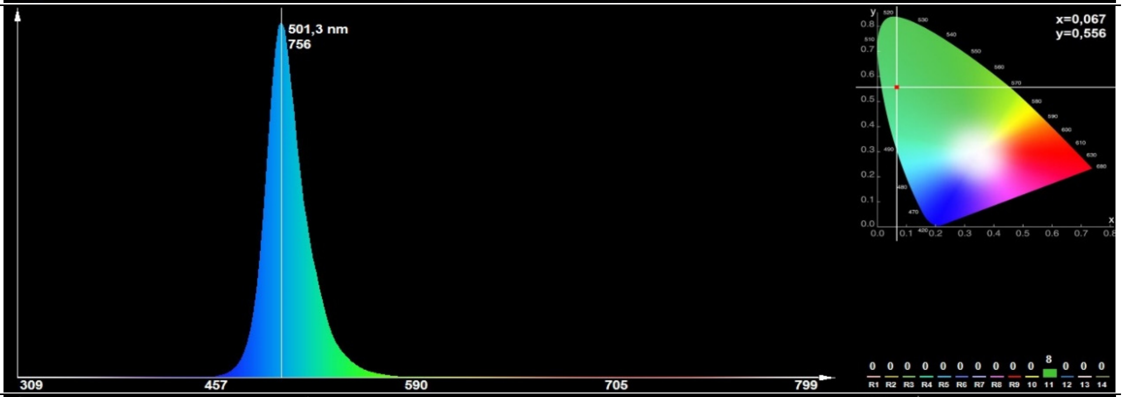

Green
525 nm

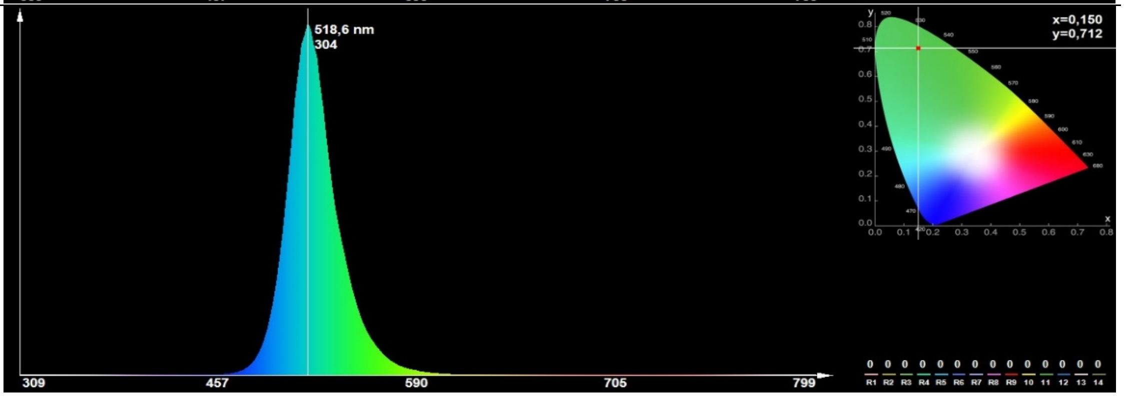

Yellow
595 nm

Red
620 - 630

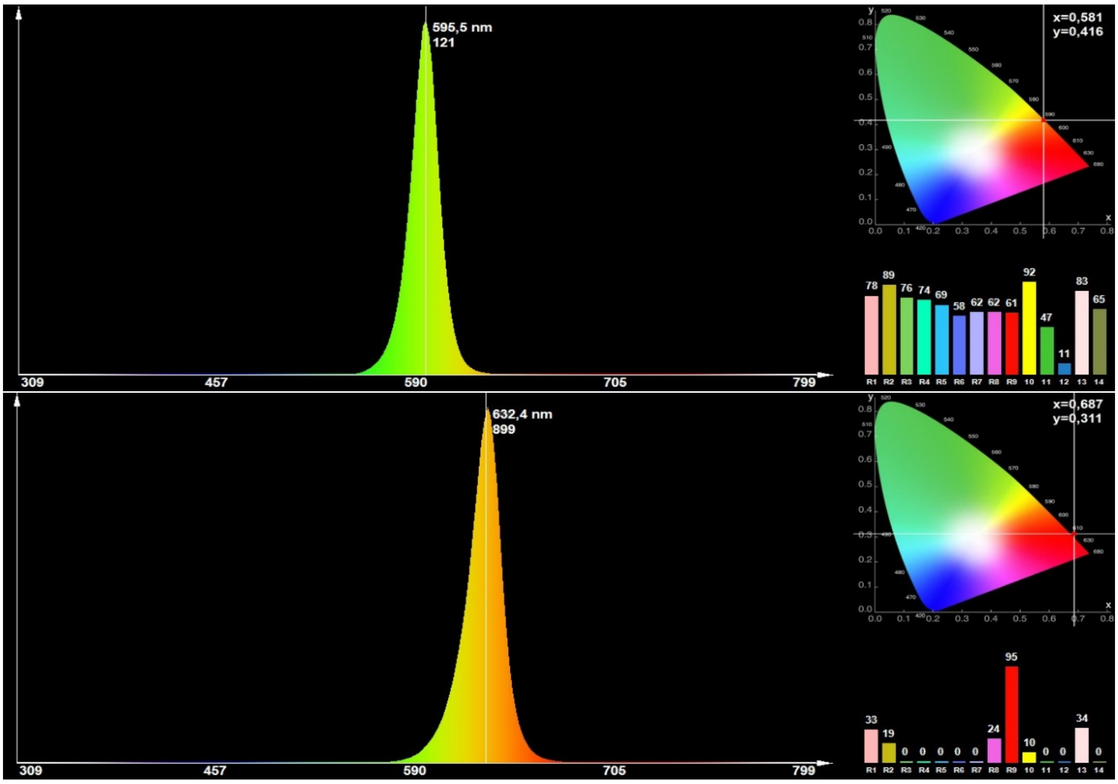

Figure S18. Emission spectra of monochromatic light sources - based on LED distributor data.
